# Recommendations for patient engagement in guideline development panels: A qualitative focus group study of guideline-naïve patients

**DOI:** 10.1371/journal.pone.0174329

**Published:** 2017-03-20

**Authors:** Melissa J. Armstrong, C. Daniel Mullins, Gary S. Gronseth, Anna R. Gagliardi

**Affiliations:** 1 Department of Neurology, University of Florida College of Medicine, Gainesville, Florida, United States of America; 2 Pharmaceutical Health Research Department, University of Maryland School of Pharmacy, Baltimore, Maryland, United States of America; 3 Department of Neurology, University of Kansas Medical Center, Kansas City, Kansas, United States of America; 4 Toronto General Research Institute, University Health Network, Toronto, Ontario, Canada; Waseda University, JAPAN

## Abstract

**Background:**

Patient and consumer engagement in clinical practice guideline development is internationally advocated, but limited research explores mechanisms for successful engagement.

**Objective:**

To investigate the perspectives of potential patient/consumer guideline representatives on topics pertaining to engagement including guideline development group composition and barriers to and facilitators of engagement.

**Setting and participants:**

Participants were guideline-naïve volunteers for programs designed to link community members to academic research with diverse ages, gender, race, and degrees of experience interacting with health care professionals.

**Methods:**

Three focus groups and one key informant interview were conducted and analyzed using a qualitative descriptive approach.

**Results:**

Participants recommended small, diverse guideline development groups engaging multiple patient/consumer stakeholders with no prior relationships with each other or professional panel members. No consensus was achieved on the ideal balance of patient/consumer and professional stakeholders. Pre-meeting reading/training and an identified contact person were described as keys to successful early engagement; skilled facilitators, understandable speech and language, and established mechanisms for soliciting patient opinions were suggested to enhance engagement at meetings.

**Conclusions:**

Most suggestions for effective patient/consumer engagement in guidelines require forethought and planning but little additional expense, making these strategies easily accessible to guideline developers desiring to achieve more meaningful patient and consumer engagement.

## Introduction

Patient and consumer engagement is now internationally recognized as an important component of clinical practice guideline development. This engagement recognizes that patients are experts, respects the rights of citizens in health policy development, empowers and informs consumers in healthcare decisions, and leads to the development of more patient-centered and trustworthy guidelines [1]. Patient and consumer engagement impacts key elements of recommendation development, including identifying whether the problem is a priority, determining if effects are meaningful, weighing risks and benefits, analyzing the impact of costs, and assessing acceptability and feasibility [2]. For these reasons, several organizations recommend or require that patients, patient representatives, or health consumers be engaged on guideline development panels alongside physicians and other professional stakeholders, including the Guidelines International Network (G-I-N) [3], the United Kingdom’s National Institute for Clinical Excellence [4], and the United States’ Institute of Medicine [5].

While there is now widespread acceptance of the value of engaging patients, scant evidence exists on whether this takes place or optimal methods by which to engage patients. A survey of guideline developers found that only 29% always involve consumers and 39% involve consumers “only if necessary” [6]. This may reflect known barriers to engaging patients and consumers in guideline development, including uncertainty of how to incorporate patient experiences into evidence-based guidelines [7], the resources needed to engage patients well [7, 8], participant feelings of isolation [8], difficulty understanding medical terminology and assessing research quality [3, 4, 7–9], recruitment difficulties [8], and inadequate training and support [4, 7, 9].

A 2006 Cochrane review found no trials investigating public involvement in guidelines and concluded that “little research has been done to find the best ways of involving consumers in healthcare decisions at the population level” [10]. There is a similar paucity of data when considering best practices for engaging patients in research settings (as opposed to guidelines) [11, 12]. Most suggestions for engaging patients and consumers stem from the practical experiences of guideline developers. These experiences suggest the importance of recruitment strategies [13], including at least two patient/consumer representatives [9], considering compensation [1], early engagement continuing throughout the guideline process [9], training [1, 4, 13, 14], support [1, 4, 9, 13], clear communication [14], clarity regarding the patient role [4], equitable treatment [14], managing group dynamics [1], skilled moderation [4], specifying guideline scope [4], making discussion of patient and caregiver issues a specific agenda item [4], and funding additional methods of soliciting patient views when needed [4].

No identified research identifies the views of patients and consumers not yet recruited to participate in guideline development. This is an important knowledge gap. While some guideline developers use experienced patients and consumers, such as those trained through Consumers United for Evidence-Based Healthcare, many developers recruit patients and patient representatives without prior training or experience. Research suggests that public awareness of guidelines is low and that individuals aware of guidelines have only a vague understanding of what they are and how they are developed [15]. Given this, and given that difficulty recruiting is one of the most frequently cited barriers to patient and public involvement in guidelines [8], investigating the views of potential participants has the potential to generate important guidance for developers seeking to engage guideline-naïve patient and consumer representatives.

Additionally, despite the above forays into highlighting practical mechanisms for patient and consumer engagement in guidelines, critical questions remain unanswered, such as the ideal balance of patients/consumers and healthcare professionals on guideline panels, composition of non-professional stakeholders (e.g. patients, caregivers, advocates, consumers), and potential practical measures for improved involvement.

Given this gap, we performed a pilot study with focus groups of individuals not involved in guideline development in preparation for a larger study on patient engagement in guideline development. The aim of the pilot study was to explore guideline-naïve individuals’ preferences for engagement in guideline development if invited, including topics relating to panel composition, barriers to successful engagement, and proposed strategies to enhance meaningful participation.

## Methods

### Approach

Qualitative focus groups were conducted to explore patient preferences for engagement in guideline development panels with physicians in preparation for a larger study. Focus groups were chosen over individual interviews to build synergy as participants listened to the views of others, prompting them to contribute additional ideas. A qualitative descriptive approach [16] was used to collect and analyze data. This technique describes straight-forward accounts of people’s views and provides a comprehensive summary without an intention to generate or test theory. This approach also allows themes to emerge inductively from the data rather than pre-specifying themes that might result in missing new ideas. After transcripts were analyzed, results were compared to an evidence-based framework for patient and consumer engagement in research in order to organize the themes within keys to successful engagement [17]. Consolidated criteria for reporting qualitative research [18] guided the reporting of study findings (S1 Checklist). Conduct of this study was approved by the University of Maryland Institutional Review Board (IRB) (HP-00065057, exempt category) and by the University of Florida IRB-02 (2015-U-1204).

### Sampling and recruitment

Purposive sampling was used to recruit Individuals through programs designed to link community members to academic research, particularly in roles relating to consultation and guiding research development (as opposed to participation as research subjects). Participants were recruited through the University of Maryland PATIENTS Program (http://patients.umaryland.edu/) and the University of Florida Citizen Scientist Program (https://www.ctsi.ufl.edu/about/ctsi-programs/implementation-science/about-the-citizen-scientist-program/). Use of these two programs provided access to patients with varying levels of experience and comfort with discussions about patient engagement. This was the first focus group for each of the PATIENTS Program participants, whereas members of the Citizen Scientist Program had met with physicians in other consultation roles. No participants had experience in guideline development and many participants were unaware of guidelines. Participants varied by age, gender, and race. Potential participants were contacted by the respective program coordinators regarding the focus group opportunity. According to different IRB recommendations, participants at the University of Maryland were not required to provide formal consent but the purpose of the focus group was explained in the recruitment call and at the beginning of the focus groups and potential participants had the opportunity to decline or leave. Participants at the University of Florida provided written informed consent for participation. Participants received a small honorarium and parking/transportation reimbursement as per the standards of the individual programs.

While focus groups were planned, one individual provided a key informant interview when she arrived after the focus group had ended (this possibility was covered under the IRB approval based on the prior experiences of the PATIENTS Program). Because participants were recruited through mechanisms attempting to increase community involvement in research planning, it was felt important to respect her attendance and contribution. Her responses were consistent with the discussions in the focus groups and so were considered alongside the other results.

### Data collection and analysis

A focus group guide (S1 File) was developed according to a review of published literature and revised in consultation with individuals involved in guideline development and health care professionals involved in the PATIENTS Program. This guide specifically queried participants’ views regarding unexplored elements of patient and consumer engagement in guidelines, such as the ideal balance of patients/consumers and healthcare professionals on guideline panels, desirable characteristics of stakeholders (e.g. relating to race, role, and prior interactions with other panel members), and anticipated barriers and facilitators to successful engagement. Specific feedback on published barriers and facilitators was not sought, preferring instead to allow participants to generate their own thoughts on what might promote or hinder their participation in a guideline group from the perspective of someone who has not yet been engaged.

Two separate University of Maryland PATIENTS Program focus groups were each led by an individual with experience in leading other focus groups with the PATIENTS Program (one white male doctoral student, one African-American female post-doctoral fellow) with one of the investigators (MJA) present. The University of Florida focus group was led by one of the investigators (MJA). Focus groups occurred on the respective campuses and lasted up to 1 hour. All groups were audio recorded with the participants’ knowledge and transcribed verbatim, therefore member checking was not employed. Qualitative analysis was performed according to a qualitative descriptive approach [16] and was used to identify, define, and organize themes. Spreadsheet and word processing programs were used to organize the data. Two investigators (MJA, ARG) independently analyzed the content of two focus groups to create a log of codes reflecting emerging themes and sample quotes illustrating theme coding (open coding). The two reviewed and discussed coding after coding each of these two focus groups to achieve consensus on emerging themes. MJA then analyzed remaining transcripts using the constant comparative technique to identify all instances of the coding framework and items not corresponding to themes in the framework and expand or merge thematic codes (axial coding). This was then reviewed by ARG. Subthemes were identified by both MJA and ARG. Themes and quotes were similar between focus groups and so groups were combined for the analysis.

Results were compared to an evidence-based framework for patient and consumer engagement in research [17]. This framework was developed from a metanarrative systematic review of publications regarding patient and public involvement in health services and medical research, with input from a patient advisory group. The framework describes four essential components of patient engagement in research: (1) patient and service user initiation (selecting the right participants and engaging them at the right time in the right way), (2) building reciprocal relationships (engaging patients as equal partners with established roles), (3) co-learning (providing opportunities for all participants to gain new skills), and (4) re-assessment and feedback. Themes identified in the current analysis were considered in light of these components and categorized within the components where applicable.

## Results

A total of 15 individuals participated over three focus groups and one key informant interview. The majority of participants were female and/or African-American with a diverse representation of ages (Table 1). Participants largely viewed themselves participating in guideline panels as patient representatives with disease-specific experience rather than as consumers, so the term “patients” is used throughout the results except where consumer involvement was specifically discussed. The focus groups at the University of Maryland each used less time than allotted; the University of Florida focus group was able to address each of the planned questions within the full allotted time. Results from the focus groups are presented here in themes emerging from the analysis.

**Table 1:** Participant demographics.

Characteristic	N (Total n = 15*)	University of Maryland–Group 1 (n = 3)	University of Maryland–Group 2 (n = 4)	University of Florida (n = 7)
Gender (female)	13 (87%)	3	3	6
Race				
Black or African American	10 (67%)	3	4	2
White	5 (33%)	0	0	5
Age				
<30 years-old	3 (20%)	0	2	1
30–40 years-old	2 (13%)*	0	1	0
40–60 years-old	4 (27%)	3	0	1
>60 years-old	6 (40%)	0	1	5
Location				
University of Maryland	8*			
University of Florida	7			

*These numbers include the key informant interview participant, who was an African American woman, 30–40 years-old

### Interest in the topic of engaging patients on guideline panels

Participants were generally interested in the focus group topic and contributing to the field of patient engagement in guideline development. As stated by one participant,

I think that it’s a positive move forward to have patients involved in guideline development… I just think it’s important to have the patient involved. How can you exclude the person that’s going through it and think that you would know everything about it? To me it just makes sense to include the patients in guideline development (#1).

### Perceived contribution

Participants said that they could contribute knowledge that was unique and complementary to that possessed by doctors:

[The doctor] may have studied something that I haven’t but I think he or she would benefit also to listen to the patient because they are experiencing it every day so there is value in both (#1).

In particular, they felt that patients can convey the bigger picture of what it’s actually like to live with the condition:

If you are living with the condition, that’s like testimony, like telling your viewpoint on what your experience has been with it (#1).

Participants highlighted that different patients may have unique perspectives and experience to contribute:

Everybody’s experience is not the same because I do feel there’s always something to learn from each. And you’ll find some common things and that’s important to know, the common stuff but it’s equally important to find the not so common stuff (#1).

Perspective… I think that’s very important especially when policy is being made… your body’s different from everybody else (#2).

They saw themselves as life experts to accompany the technical and educational expertise of physicians:

You know I can respect the doctor as the expert on the technical side or educational side but I want him to respect me as the expert as the patient living (#1).

### Ideal characteristics of guideline development groups

#### Panel composition: Number of patients to physicians

One area of considerable differences of opinion was regarding the optimal number of patients and physicians on the panel, particularly the ratio of the two (S1 Table). Some participants felt that they would be comfortable as the only patient representative on a panel, whereas others said this would make them feel like they didn’t belong and would be intimidating. Those who expressed feeling more comfortable with the idea of serving as the sole patient representative were older and described more experience with either physicians or group dynamics.

While some participants stated that they would feel comfortable as the only patient representative, there was general consensus that including multiple patient representatives would be ideal (S1 Table). Some participants indicated that simply having multiple patient participants would be enough for them to feel comfortable, while others indicated that they would want there to be an equal number of patients and physicians:

If I was in a group like that I would want to have peers to feel comfortable and I would also want to have probably a balance between people like physicians, authorities (#3).

Several participants advocated that the majority of guideline panel members should be patients, with physicians constituting the minority of participants (S1 Table). Regardless of panel composition ratio, participants endorsed that smaller panels would make them more comfortable presenting their views, particularly to physician panelists (S1 Table).

#### Characteristics of patients/consumers on guideline development groups

The Citizen Scientist Program focus group comprised of individuals with prior experience working in an advisory panel setting unrelated to guidelines had opinions about the characteristics of individuals that should be included on panels (S1 Table). They recommended that patient panelists should be knowledgeable and unbiased, have relevant expertise, and be willing to actively contribute and ask questions. In each focus group, there was broad consensus that participants would be comfortable participating on a panel with other patients they had never met before, with some participants indicating that they would feel more comfortable sharing opinions in front of strangers (S1 Table).

Most respondents said they would feel comfortable on panels including both patients and caregivers, whether considering this from a patient or a caregiver perspective, but one individual disagreed with this, saying that he would be less likely to share as a patient if there was a caregiver in the room and that he would feel like he was an “intruder” if he was a caregiver.

Some participants suggested that including an advocate could be helpful to represent a wide variety of individuals living with a disease and to serve as a mediator on the panel between physicians and patients (S1 Table). While participants focused on the value of patient representatives on guideline panels as contributing life experience, some also described that involving individuals without specific disease experience (i.e., consumers) could bring different perspectives and additional value:

If we’re talking about a specific disease or something like that, it probably would be helpful to have representing the patient side somebody that isn’t in that disease group just to give a kind of a…you know an outsider’s perspective… (#3).

It was noted that this person could also represent the patient experience more generally:

[They can] set a frame work for the type of questions that patients tend to ask (#3).

At the same time, some participants felt that having some content knowledge would be beneficial:

As consumers… we have to have some knowledge of what is stage 1, what is stage 2, what is stage 3… (#3).

#### Characteristics of healthcare professionals on guideline development groups

Views were mixed about whether patients would be comfortable if their own physician was on the panel. Participants who indicated that they had a good relationship with their personal physicians were more likely to express comfort in participating on a panel with their personal physicians. Several individuals expressed discomfort at the idea of participating with their personal physician, fearing loss of confidentiality or judgment regarding their opinions (S1 Table).

Most participants indicated comfort with the idea of participating on a panel with physicians that they would be meeting for the first time, but a couple participants indicated that it would be difficult to learn to trust physician participants, particularly if credentials were unknown (S1 Table).

Most participants said that physician race or ethnicity would not matter, but several African-American participants indicated that they would be more comfortable if there was a physician of the same race on the panel:

I would think that, I’m going to probably gravitate to the person of the same cultural race, you know it would be like, you know what I mean, you know to even interrupt you know and just to feel comfortable and a little more trusting of the information given from the physician, you know…yeah that’s big (#2).

Another individual emphasized the importance of diversity in general:

I’m a great advocate for diversity. I don’t want to hear opinions of one particular group of people (#3).

Several participants valued having a variety of health professionals on the panel (e.g. social workers and pharmacists in addition to physicians) to include different perspectives in the discussion and provide additional support to patients (S1 Table).

### Perceived barriers to successful patient participation on guideline development groups

Participants identified potential barriers to successful participation including spoken language (e.g. difficulty understanding accents), medical terminology, possible physician resistance to patient participation, personal lack of content knowledge that would make them less likely to share an opinion, group dynamics, and insufficient time for discussion (Table 2).

**Table 2:** Perceived barriers to successful patient participation on guideline development groups.

Barrier	Exemplary Quotes
Spoken language	• If physicians were foreigners [it could be more difficult], because sometimes they don’t speak the same way that Americans speak, some people talk more with slang, so I would watch how I word stuff in the room with people from other countries (single). • I think one of the difficulties is we’re getting a huge bunch of individuals with accents dealing with patients that the patients are finding it very hard to understand and follow (#3).
Medical terminology	• Sometimes physicians can speak in terms that we may not be familiar with (#1). • A lot of times doctors tend to talk in technical and clinical terms so I would, maybe need somebody to help translate, you know (#2). • … not having a whole array of doctors that are going to get into a language that will be very confusing and would intimidate the participants not to say anything (#3).
Anticipated physician resistance to lay involvement	• But I can imagine that would be a little bit…threatening [to physicians]… it would uh…what are we…we’re the experts here, we know what we’re doing so is there likely to be resistance to inclusion of the lay community in this? (#3).
Lack of content knowledge	• If it’s a topic that… I may not know that much about, that would keep me quiet because then I’m in my listening mode (#1). • Unless you feel a little bit educated in the area at least somewhat, you don’t want to speak up (#3).
Group dynamics	• You don’t want to come off offensive… you don’t want to create tension between you and another person (#2). • Just the basic group dynamics. If you’ve got a really strong person who takes over everything then that’s really going to turn you off, you’ll never want to go back (#3). • If you have someone who’s not participating at all, that’s equally frustrating, so the character of the group is important (#3).
Insufficient time	• We had one of these group discussions and really we didn’t have much of it because there was too much presentation and very little time for input and we each kind of quickly went around the table, there’s no opportunity for follow up and so forth, so there has to be sufficient time… the larger the group the more time you need (#3).

### Recommendations for successful engagement

Participants volunteered a number of ideas on how to make patient engagement on guideline panels more successful (S2 Table), starting with establishing the purpose of the panels and what they need to accomplish:

Make your purpose of the group clear. Like, “This is what we’re here to do, this is why you are here, this is the overall goal, why this is important.” I feel like everyone in the group will have somewhat of an understanding of why they are there and what their purpose is and what they are doing and that’s very important (#2).

Participants also expressed an interest in knowing the guideline topic and receiving pre-reading to prepare and start forming opinions. They suggested identifying a point of contact within the guideline organization for communications like pre-meeting clarification. There was a general sense that email was the preferred route of communication but participants suggested that guideline developers should know the communication preferences of individual participants and be willing to target strategies to those preferences. They warned that developers should be cautious not to overwhelm panel members with communications.

Several participants stressed the importance of having a skilled facilitator who is sensitive to the challenges of leading a group of physicians and patients, able to make sure the patient voice is heard, and skilled at moderating:

It would seem to me to be important whoever leads the discussion has to have a great deal of sensitivity because…I can see where there would be a tendency to overwhelm the lay part of the audience (#3).

That person would have to be really carefully selected it would seem to me to ensure that the lay part of the group gets full measure because experts in these fields are going to tend to want to lock horns, or at least talk to one another that way and that could drown out the voices of the lay (#3).

Most focus group participants indicated that they would be comfortable volunteering their opinions and would not be deterred if their opinion was different from other panel members, whether physicians or patients. Opinions differed on comfort with direct questioning by a facilitator; some participants indicated that this would be no problem while one thought this would be intimidating. Multiple individuals mentioned the advantage of having an established process for making sure that everyone has a chance to share an opinion (S2 Table). One individual who was less confident about sharing opinions wanted the ability to pass on questions or submit opinions in written format. A practical suggestion was to supply paper and pens at in-person meetings so that patients can note thoughts if there is not an immediate opportunity to share them. Some participants suggested that multiple meetings are helpful to build group dynamics but admitted that this can be logistically challenging (S2 Table).

Finally, participants suggested that guideline developers have a mechanism to make sure that the patient voice was incorporated:

If that’s the case [resistance from physician panel] then…in the review of the results coming from the discussing, somebody or probably more than one person, has to look at the results very carefully and see if the voice of the lay community was not heard as it might have been (#3).

### Context within a framework of patient and service user engagement in research

**Fig 1:**
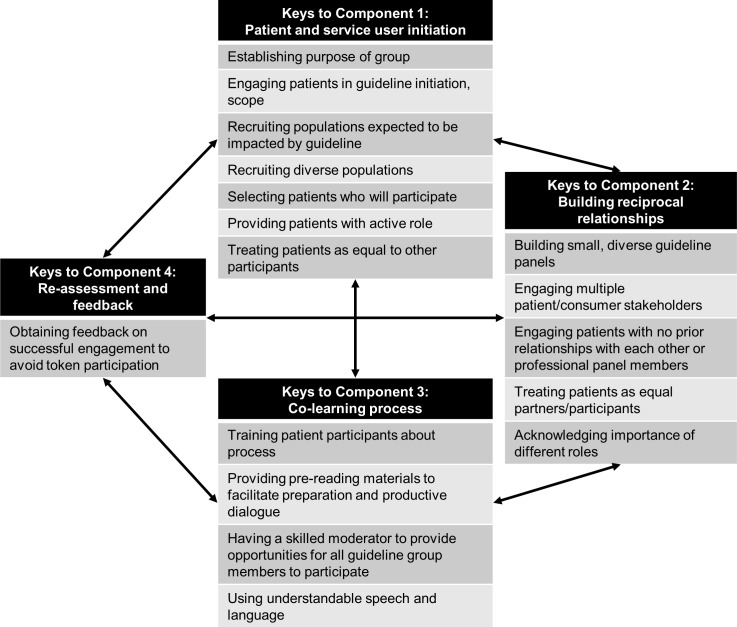
Key themes as they relate to integral components of patient engagement.

Several identified themes fit within Component 1 of the framework for patient engagement in research–patient and service user initiation–describing recruiting patients into the process (Fig 1). Focus group participants highlighted the importance of establishing the purpose of the group up front; other publications have also emphasized the importance of engaging patient stakeholders from the very start of guideline initiation and scoping [4, 9]. Diversity is important with regards to recruiting individuals with different disease perspectives, ethnicities, and roles. Selecting participants who will actively engage is also vital.

Providing patient representatives with an active and equal role is key to both Component 1 and Component 2 of the framework, where Component 2 focuses on building reciprocal relationships. Additional keys to Component 2 identified within these focus groups include designing small guideline panels with multiple patient/consumer stakeholders and engaging patients with no prior relationships with one another or the professionals on the panel. Many of these strategies aim to enable patients’ power within the process, acknowledging the unique importance of their role. This is also accomplished through Component 3 –co-learning–which prevents professionals from dominating the process. Themes key to this component include training, preparatory materials, skilled moderation, and use of accessible language. Finally, focus group participants highlighted the importance of assessment to make sure that patient engagement was not merely tokenistic and this is reflected in Component 4 (Fig 1).

One theme not fully captured in the referenced framework is that of practical support. The framework focuses on the relational components of patient engagement, but focus group participants had numerous suggestions for how meeting logistics and practical support could facilitate engagement. Consistent with the bidirectional relationships between the four framework components, this additional consideration–support and logistics–overlaps with the other components. Support and logistics contribute to user initiation by providing a point of contact and information regarding guideline development and expected roles. Providing adequate time, considering multiple meetings, and pre-specifying mechanisms of soliciting opinions enable building reciprocal relationships. Providing pre-reading materials facilitates co-learning, as does allotting adequate time and providing physical resources (e.g. for note-taking). Methods of practical support allow re-assessment and feedback, which can also inform if other strategies would be helpful in the future. The need for initiation, reciprocal relationships, and co-learning informs support and logistics, further emphasizing the model’s bidirectional nature. The numerous practical suggestions from participants suggest that an additional support and logistics component to the framework could be considered.

## Discussion

This study suggests that ideal guideline panels are small and engage multiple patient/consumer stakeholders (Table 3). Diversity is important with regards to engaging individuals with different disease perspectives, ethnicities, and roles (patients, consumers, advocates, and healthcare professionals of various backgrounds). Given that some participants described discomfort with certain situations (e.g. mixing patients and caregivers on a panel), potential participants should know anticipated panel composition in advance. In general, the focus groups preferred anonymity. To facilitate patient/consumer engagement, guideline developers should ensure that meetings are conducted using language that all can understand and with sufficient time allotted for sharing opinions and discussion. Providing writing materials is a practical way to help panel members note thoughts to share. When recruiting patients and consumers, guideline developers should establish the purpose of their engagement and provide preparatory materials and a point of contact. Facilitators should be sensitive, skilled moderators, and able to engage all panel members, possibly with pre-established plans for soliciting views, particularly if panel members are hesitant to volunteer opinions. Multiple in-person meetings may build comfort but have logistical challenges.

**Table 3:** Key Suggestions for Guideline Developers.

Guideline Step	Key Suggestions
Recruitment	• Provide information regarding guideline development to potential participants to allow a more informed decision regarding participation • Involve multiple patient stakeholders who don’t know each other or the professionals on the panel • Engage diverse patients (e.g. race, age, disease experience) • Consider consumer stakeholders in addition to patients • Recruit non-patient, non-physician stakeholders (e.g. social workers) • Target small guideline groups • Recruit diverse physician/professional participants (e.g. race, age, experience) • Choose a skilled moderator
Preparation	• Provide a point of contact for participants • Provide pre-participation training • Provide reading materials so that participants can prepare in advance • Identified preferred mechanisms of contact (e.g. email, phone) • Limit contact to essential communications
Logistics/Conduct	• Plan adequate time • Pre-specify methods for providing patients opportunities to share opinions • Provide physical resources (e.g. pens, paper) to facilitate involvement • Ensure that discussion is conducted in understandable language • Consider multiple in-person meetings
Re-assessment	• Establish mechanisms for reviewing impact of patient engagement to avoid tokenistic engagement

These results are consistent with prior publications. Various groups recommend multiple patient/consumer stakeholders [4, 8, 9, 19]; isolation is a barrier to successful patient engagement [8]. Diversity in panel composition is also recommended by the Institute of Medicine [5], G-I-N [3], and AGREE2 [20]. Medical terminology is a well-established barrier to successful patient engagement [7–9] and was also highlighted in our study. Perceived barriers of physician resistance to patient participation and personal lack of content knowledge are consistent with known barriers [8]. Establishing the purpose of patient participation in guideline panels is described as a facilitator of successful engagement in other reports [4, 8]; providing pre-reading materials can address pre-meeting apprehension [4] and can serve as one method of advanced training to facilitate engagement [4, 8]. Use of a “welcome pack” is described as a facilitator to successful patient engagement and is a method of providing both training and support [8]. The importance of engaging a skilled chair/moderator is a point made by our participants and others [1, 4, 8].

New implications include an emphasis on the logistical and practical measures that guideline developers can take to enhance patient engagement (Table 3), something not captured in a recent patient engagement framework. Additionally, to our knowledge, this study is the first to explore the preferences regarding pre-meeting relationships of panel members and issues regarding race and diversity. While medical terminology is a well-established barrier to successful patient engagement, the barrier of difficulty understanding spoken language (i.e., accents) was also identified by multiple participants. Lack of sufficient time for meetings/collaboration is a newly identified (though hardly surprising) barrier that developers can address. Finally, study participants made the point that guideline developers and panels should assess panel results and products to ensure that the patient voice was adequately captured and avoid tokenistic engagement.

While not directly suggested by focus group participants, a strategy implied by the findings is the importance of educating potential participants *prior* to their agreement to join a guideline development group (Table 3). This makes sense given limited public knowledge regarding guidelines and guideline development [15, 21]. In the guideline-naïve participants in this study, there were misconceptions about guidelines and the guideline process such that some suggestions were impractical (such as patients greatly outnumbering professional participants on a panel). This suggests that potential participants may not understand the process, goals, and scope of involvement and this should be clarified both before and after consent to participate. Additionally, given that some participants described discomfort with certain situations (e.g. mixing patients and caregivers on a panel), potential participants should know anticipated panel composition in advance. This advanced education is in addition to training individuals who have already agreed to participate, a well-described facilitator to engagement [1, 3, 4, 8, 13].

Limitations of the present study include the fact that participants had no experience with guideline development and minimal knowledge of guidelines prior to participating in the focus groups. Thus, they were making judgments and recommendations based on hypothetical engagement. However, developers recruiting patients for guideline panels are likely to largely encounter patients or members of the public with no knowledge of guidelines, making these findings particularly helpful for developers preparing to promote and support the involvement of guideline-naïve patients/public in the process. It is notable that many comments and suggestions by our guideline-naïve participants reflect barriers and facilitators described in the literature by those with guideline expertise. This underscores the power of obtaining the patient view–these participants were able to identify in advance barriers and facilitators to engagement that developers are learning by reflecting on initial experiences. Naïve statements and an identified lack of understanding regarding some aspects of guideline development also informed some key suggestions (Table 3), such as training as part of recruitment rather than solely after an agreement to participate.

The lack of understanding reflected in some responses could also result from the description of guideline development at the start of the focus groups (S1 File), which was intentionally kept simple to enhance understanding by participants with varying health literacy. Guideline methodologists and participants drafted and revised outline content and edits to enhance patient and consumer understanding were implement by PATIENTS Program staff, but the introduction did not capture all nuances of guideline development nor the breadth of different developers’ approaches. Given the overlap between our results and those in the literature, participants appeared to understand many aspects of guideline development based on the introductory material. Some impractical suggestions, such as patients and consumers greatly outnumbering professionals on guideline panels, persisted even after clarification of typical guideline group composition (e.g. professionals representing different areas of expertise, such as content vs methodology expertise). Thus, these unexpected views are important for guideline developers to consider, both in terms of adequate engagement and recruitment training.

We did not solicit feedback on published barriers and facilitators to patient engagement, preferring instead to allow participants to generate their own thoughts, particularly as we were investigating the views of guideline-naïve potential participants (in contrast to currently published literature). This means that some well-accepted facilitators to engagement, such as training, were not explored in our focus groups. These focus groups reflect a pilot study preparing for a larger project investigating the impact of engaging patients, caregivers, and advocates on guideline question development and as such, only two patient organizations were targeted. This means that saturation of themes may not have been achieved. Participants agreed to one-time group participation and not future contact, so feedback on transcripts and themes was not obtained from participants subsequent to the focus groups. Finally, while attempts were made to obtain diversity in age, gender, and ethnicity, the number of participants and focus group conduct in two locations in the United States means that these findings are potentially not transferable to patients in other locations.

Future research should further explore the implications of panel composition including issues of race and racial diversity, the impact of engaging non-physician professionals, and the optimal balance of lay and professional participants. Research is also warranted into logistical issues, such as the impact of meeting type (in-person, telephone, webinar, all-electronic) and frequency. Understanding optimal strategies for engagement will require further insight into these practical considerations in addition to the topics of strategic recruitment, relationship-building, co-learning, and reassessment. It is also important to understand if certain factors have more impact on successful engagement such that they should be prioritized by guideline developers. Finally, future research could directly compare views of participants with and without guideline experience or investigate views of guideline-naïve individuals before and after guideline development group participation to further understand how views differ between those individuals with and without guideline experience.

## Conclusions

This study adds an additional voice to the research regarding patient engagement in guideline development panels: that of individuals not yet recruited. Emerging recommendations for successful engagement overlap with existing literature but also provide new strategies, including highlighting the importance of practical and logistical issues. Guideline developers should consider small, diverse panels with multiple patient/consumer stakeholders having no prior relationships. Providing goals for participation, pre-meeting reading/training and a point of contact are keys to successful early engagement; skilled facilitators, an emphasis on understandable speech and language, and established mechanisms for soliciting patient opinions enhance patient engagement at meetings. Most of these strategies are inexpensive, an important consideration for many guideline developers.

## Supporting information

S1 Checklist COREQ Checklist. (DOCX)

S1 File Focus Group Outline and Possible Prompts. (DOCX)

S1 Table Ideal characteristics of guideline development groups: illustrative quotes. (DOCX)

S2 Table Recommendations for successful engagement of patients on guideline development panels: illustrative quotes. (DOCX)
