# Supplementary material for: Recommendations for patient engagement in guideline development panels: A qualitative focus group study of guideline-naïve patients
Source: PLoS One. 2017 Mar 20;12(3):e0174329. doi: 10.1371/journal.pone.0174329 (PMC5358846; doi:10.1371/journal.pone.0174329)
Supplement: S1 File — (DOCX) [file pone.0174329.s002.docx]

Supplement 2. Focus Group Outline and Possible Prompts

Thank you for coming today to participate in this focus group. We are planning a study to look at the impact of partnering with patients, caregivers, and patient advocates to develop “clinical practice guidelines.”

Guidelines are summaries of what we know about the usefulness and safety of different tests and treatments for health problems. Guidelines are based on research, but there are steps in developing guidelines where experience and opinions matter. The first step in developing guidelines is deciding what questions are important to ask. It is possible that physicians, patients, and families will have different views on what health questions are the most important questions to ask. We are interested in looking at how partnering with patients, family members, and patient advocates in guidelines might change the guideline questions.

In order to develop guidelines, groups of 6-8 individuals work together to decide what questions to ask. Then some of these individuals look at all the research available to answer the questions and grade the strength of the research. After looking at the research, the guideline group then makes recommendations on what we know (or don’t know) about the best medical care. Because each person has unique health, circumstances, and preferences, guidelines do *not* tell doctors the best way to treat any individual patient. They provide information about the amount and quality of the research available to support a certain medical decision.

For example, one guideline^1^ strongly recommends that doctors encourage men between the ages of 45 and 79 to take an aspirin every day to prevent heart attacks, as long as they don’t have a high risk of stomach bleeding from the aspirin. The same guideline points out that we don’t have enough research to know the benefits and risks of daily aspirin in people 80 years and older, so it makes no recommendations about what to do for people this age. It is important for guidelines to point out the times when we know the best treatment and the times when there is not enough research to know the best thing to do.

*Optional additional example:*

*In a different example, the American Academy of Neurology tells doctors that they should “probably” use blood thinners in people with irregular heartbeats and a history of stroke to try to prevent further strokes.^2^ The recommendation says “probably” because the strength of the research is moderate, not strong. The American Academy of Neurology also point out that there are important reasons that some people can’t take blood thinners.*

For each of these guidelines, groups started by asking questions. For example, the first guideline group asked, “For men at different ages, does taking a daily aspirin help prevent heart attacks without increasing bleeding risk too much?” The guideline group felt it was important to know about both the way aspirin could help (decreasing heart attacks) and the risk (bleeding). The second guideline group asked, “For people with an irregular heartbeat and a stroke, does a blood thinner help prevent another stroke?”

As mentioned at the beginning, we want to learn how partnering with patients, family members, and patient advocates in the development of guidelines might change the guideline questions. To do this, we will host guideline groups in Minneapolis next year, one with just physicians and one including physicians, patients, caregivers, and patient advocates. Before we start those groups, we want to know if you have opinions on how you would want to be involved in a guideline group if you were asked. Your input today will help set the tone for the guideline groups next year and will help us make these guideline groups successful.

Do you have any initial questions or thoughts?

*References in case someone asks:*

*(1) US Preventive Services Task Force: Aspirin for the Prevention of Cardiovascular Disease, Grade A and I (insufficient)*

*(2) AAN Prevention of stroke in non-valvular atrial fibrillation (Level B)*

Possible questions for discussion (to be asked as appropriate in the conversation; need not be asked in this order):

***Let’s talk about how the guideline group would look:***

1. Many current guideline groups only include physicians. If you were a patient or a family member in a guideline group, how many patients and/or family members would need to be in the group for you to feel comfortable and why (give reasons)?
   1. How comfortable would you be in a group where you were the *only* person who was not a physician? (Give reasons.)
   2. Would the number of the physicians in the group affect how comfortable you would be? *(Prompts: How would you feel if there were 3-4 physicians? 5-7 physicians? 8-10 physicians? Explain reasons.)*
2. Often people in a guideline group do not know each other. How would you feel about being in a guideline group with people that you do not know? *(Probes: Would you be more comfortable if you knew the other patients or family members? Would you be more comfortable if you had never met them before? Consider following up with questions about being from same/different neighborhoods, having same/different medical conditions.)*
3. Sometimes people with poor health or limited abilities due to health problems need caregivers. As a patient, how would you feel about being in a group including a caregiver of someone with the same condition? *(Probes: “For example, if you had a stroke and needed help because of weakness, would you be comfortable being in a guideline group for stroke where one of the other people in the group is a wife caring for her husband with a stroke?*

*OR: For example, if you have severe depression, would you be comfortable being in a guideline group about depression where one of the other people in the group is a husband who helps take care of his wife with depression?)*

- 1. As a caregiver, how would you feel about being in a group including a patient with the same condition as the person you care for?

***Let’s talk about the physicians in the guideline group:***

1. Tell us how would you feel if you were in a guideline group with
   1. your personal physician?
   2. physicians you’ve never met before?
2. How do think the race or ethnicity of the physicians on the panel would affect your willingness to make suggestions?

***I’d like to know more about how you view your participation in the guideline group:***

1. How comfortable would you be spontaneously offering an opinion in a guideline group? Please explain.
2. What would you do if you had a different opinion from other guideline group members?? *(Probes: How would you handle disagreements/different opinions with other patients/family members? With physician group members?)*
3. How would you feel if the group leader directly asked you for your opinion during the discussion?
4. What do you think might cause you to limit what you say in the group? (Probe: Certain people in the room, lack of trust, etc.)
5. What things do you think we can do to make it easier for you and other patients/caregivers to participate and contribute in the group?
6. What do you hope patients might be able to add by participating in a guideline group?

If focus group includes older individuals and/or individuals with experience with people with memory problems/dementia:

*We are interested in involving patients, caregivers, and patient advocates in a guideline group looking at ways to diagnose memory problems.*

1. If your memory is normal, how would you feel about working in a guideline group about memory with a person with memory problems?
2. If a guideline group includes a person with memory problems, how do you think group leaders can make sure to include that person’s opinions even though the memory problems might make it harder for him or her to participate?
3. What do you think are the pros and cons of involving people with memory problems and/or their family members in a guideline group on a memory topic?
