# Supplementary material for: Recommendations for patient engagement in guideline development panels: A qualitative focus group study of guideline-naïve patients
Source: PLoS One. 2017 Mar 20;12(3):e0174329. doi: 10.1371/journal.pone.0174329 (PMC5358846; doi:10.1371/journal.pone.0174329)
Supplement: S1 Table — (DOCX) [file pone.0174329.s003.docx]

| Supplement 1 Table. Ideal characteristics of guideline development groups: illustrative quotes |
| --- |
| *Theme: Panel composition: number of patients and physicians* |
| Subtheme: Serving as the only patient representative   - I would feel… like I didn’t belong (single). - I would be fine with it (#1). - I would still speak out and give my opinion (#1). - I would be comfortable because I’ve had a similar experience (#2). - I’m pretty used to being the only one in a group (#2). - I might feel a little intimidated… They are experts and it’s my opinion so I wouldn’t think that it’s as important or because I don’t have the background of saying “hey this is wrong” or “this is how I feel.” So it might be a little hard.” (#2). |
| Subtheme: Ideal number of patients   - Just two or three… It don’t have to be a large group for me to comfortable (single) - An equal number of each population represented (#1) - If I was in a group like that I would want to have peers to feel comfortable and I would also want to have probably a balance between people like physicians, authorities (#3) - I would say if you have 6-8 physicians, I would want to have 3-5 lay people… (#3) - [Disagreement] I think it would be a little more intimidating if there’s only like 3-5 (#3) - I think fewer doctors with… maybe a larger group, maybe 4 doctors to 8 or 12 people that are a similar level of illness in the disease (#3) |
| Subtheme: Overall group size   - It needs to be smaller and kind of more personal, smaller with the doctors, patients it’s good, but the doctors can be a little overwhelming to patients (#3). - A small group to promote dialogue… 8 people maximum would be really helpful because I wouldn’t want too many… I mean I’d want to feel comfortable presenting my views (#3). |
| *Theme: Characteristics of patients/consumers on guideline development groups* |
| Subtheme: Willingness to actively contribute   - I feel like among the community there’s…people have different aspects of willingness to speak up and speak what their opinion actually are. Some people just sit there and like think all the opinions in their head and not share it (#3). - I would actually look for someone who is not a “yes” person, somebody who is not intimidated if their question is not up here… I would want somebody who will say “Ok, stop, what does that mean. Tell me” (#3). |
| Subtheme: Knowledgeable   - They are obviously well informed and you know and they have a good base to start from, they’re not just off of the street (#3). - Having a group of well informed, non-professionals working with a small group of professionals would give you a good outcome (#3). |
| Subtheme: Unbiased   - Somebody that comes in with a real axe to grind or one, single mindedness issue that they are dealing with might be counter-productive (#3). |
| Subtheme: Relevant expertise [see also perceived contribution]   - You can’t bring high school students to talk about college student topics, they are not going to feel comfortable. If you bring high school students to talk about high school topics, it’s just about what they’re going to be talking about (#2) |
| Subtheme: Participating with strangers vs people they know  *Consensus that participating with strangers is not a problem:*   - I wouldn’t mind it. It wouldn’t really matter to me (single). - I wouldn’t have a problem with that. I would be interested to hear what other people’s views are on whatever we’re talking about (#1). - I’d feel comfortable because, you know, we’re just human, we’re equal (#2).   *Concern that knowing participants would limit what one might say:*   - if it’s with people that you don’t know you may be more free to express how you feel versus when you do know you might be a little more guarded in what you say (#1). |
| Subtheme: Mixing patients and caregivers   - We’re all on the same level, the same page, talking about the same illness or whatever. We have something in common so yes I would feel comfortable (#1). - When I think anytime you’re exposed to someone else who’s going through something similar to what you’re going through, if your mind is open and receptive you’re going to get something out of that (#2). - I would be comfortable because I would feel like I’m giving my input on what I’m experiencing and hearing what they have to say as people who experience it (single). - I would feel a little guarded [as a patient with a caregiver in the room]… I would be a little apprehensive maybe to go into greater detail about my condition… (#2). - [As a caregiver in a room with patients] I would feel almost like an intruder … Because like it’s health issues of people are very personal (#2). |
| Subtheme: Use of an advocate   - And I propose that if would be beautiful to see a panel of doctors talk to a panel… of “boots support” people that are holding meetings with patients… because the people running these support groups are getting these questions that the doctors are hoping that they will get from the patients and their caregivers (#3). - I would want an ally like a social worker in there with me… because what I’m always thinking [is that] not everyone can self-advocate, you know, and a lot of times doctors tend to talk in technical and clinical terms so I would, maybe need somebody to help translate, you know (#2). |
| Subtheme: Role of consumer   - You’ll get something different from those who know [about the topic], I think you’ll get something different from people who just show up (#1). - If we’re talking about a specific disease or something like that, it probably would be helpful to have representing the patient side somebody that isn’t in that disease group just to give a kind of a…you know an outsider’s perspective… (#3). - [They can] set a frame work for the type of questions that patients tend to ask (#3). - All right but if as consumers… we have to have some knowledge of what is stage 1, what is stage 2, what is stage 3… (#3). |
| *Theme: Characteristics of health care professionals on guideline development groups* |
| Subtheme: Presence of personal physician on panel   - Pretty good because that’s my doctor, I would like that. That’s the person that I normally see and I would like for them to be there to hear what I have to say and I’m hearing everything that they have to say (single). - I would not want my personal physician there because I feel like there’s more judgment and they may change the way that they treat me or how much they respect me and try to care for me (#3). - Refer back to confidentiality. [What if] my personal physician goes, “Oh, well my patient right here has had some experience;” uses me as an example. And not everybody wants to be used as an example (#2). |
| Subtheme: Interacting with physicians they’ve never met   - I would be find with that because I’m sure they would be knowledgeable about what we are discussing (#1). - I would feel somewhat skeptical… and a little intimidated… I’d be less trusting… I think I’d want to check credentials, like “are you sure?” I mean I’m sitting in a room with all these people I don’t know, how do I know that you’re who you say you are and I don’t know (#2). - I don’t know you so how can I trust you (#2). |
| Subtheme: Race/ethnicity of physicians   - As long as they are knowledgeable, as long as they care about me (#1). - I’m not going to go through all this rigmarole where I come from, where you come from; right here, right now you are a professional and I’ll take you at your word (#2). - I would think that, I’m going to probably gravitate to the person of the same cultural race, you know it would be like, you know what I mean, you know to even interrupt you know and just to feel comfortable and a little more trusting of the information given from the physician, you know…yeah that’s big (#2). - I’m a great advocate for diversity. I don’t want to hear opinions of one particular group of people (#3). |
| Subtheme: Involving non-physicians   - There just needs to be a variety of people’s opinions so that’s why you need the patients and the advocates and the doctors and the nurses and the nutritionists, or whatever, opinion. Different opinions (#1). - I think a holistic approach is important and I think that other disciplines other than medical, like medical doctors, like social workers, um, pharmacist or whoever, uh, should be included (#3). |
