# Supplementary material for: Recommendations for patient engagement in guideline development panels: A qualitative focus group study of guideline-naïve patients
Source: PLoS One. 2017 Mar 20;12(3):e0174329. doi: 10.1371/journal.pone.0174329 (PMC5358846; doi:10.1371/journal.pone.0174329)
Supplement: S2 Table — (DOCX) [file pone.0174329.s004.docx]

| Supplement 2 Table. Recommendations for successful engagement of patients on guideline development panels: illustrative quotes |
| --- |
| **Theme: Establish purpose** |
| - I think giving people some history and background is very helpful so you will have, for me I know why I am here and then I can structure my responses to what the purpose that we are here and I’m not talking about off the wall stuff (#1). - Make your purpose of the group clear. Like, “This is what we’re here to do, this is why you are here, this is the overall goal, why this is important.” I feel like everyone in the group will have somewhat of an understanding of why they are there and what their purpose is and what they are doing and that’s very important (#2). |
| **Theme: Advance preparation** |
| Subtheme: Provide background information in advance   - They might want to write down some questions before they even come (single). - What worked well for us, is we would get documentation or wanted documentation prior to any of the meetings or sessions that we would attend… we would look at that prior to coming in, we would make notes to ourselves, you know around the sheet, well what is this, what does this mean, you know all the little finite details (#3). - If you’re going to come into the group with specific questions… if you could actually send those questions out and materials or maybe not the specific questions, but like a lot of professors will do like there’s going to be questions similar to this, you know on the exam so that we can kind of already start thinking along those lines (#3). |
| Subtheme: Provide point of contact   - Let’s say I get the materials and I have questions ahead of time, if there was a contact person that could clarify anything that’s in the written material, one could come into the actual meeting a little better prepared and not waste time up front (#3) |
| Subtheme: Mechanism and frequency of contact   - Know your audience and… people’s communication preferences (#2). - If it’s my email it comes up on my phone and I have instant access; if you send it to me in the mail, I may not check my stuff (#2). - Please don’t dumb rush me on the telephone… You can leave a message, but 1 please, not 20 messages (#2). |
| **Theme: Picking a skilled facilitator** |
| Subtheme: Sensitivity   - It would seem to me to be important whoever leads the discussion has to have a great deal of sensitivity because…I can see where there would be a tendency to overwhelm the lay part of the audience (#3) |
| Subtheme: Ensuring patient voice is heard   - Well I think as a group leader you should be able to recognize that [a person is hesitant to speak] and then present opportunities for that person to speak and that’s knowing your group (#1). - That person would have to be really carefully selected it would seem to me to ensure that the lay part of the group gets full measure because experts in these fields are going to tend to want to lock horns, or at least talk to one another that way and that could drown out the voices of the lay (#3). |
| Subtheme: Skilled moderator   - There are professional groups out there that lead these kind of groups and whoever is moderating and drawing the group out is the important person to set the stage for people being involved (#3). |
| **Theme: Communication**  Subtheme: Volunteering opinions   - I would be comfortable because I would want everybody to know my thoughts. Me, I communicate everything that’s going on with me and I also ask a lot of questions (single). - Well the whole point that we were there, I know that I’m going in with you… to know my thoughts, then I would go in with that mindset that I just can’t sit there and be a frog on the log I have to express my thoughts (#1). - Should be comfortable because everybody is opinionated, everybody is entitled to their own opinion. So why not share your opinion? Your opinion might enlighten somebody else (#2). - I would be fine with it, but I know that when I first came to this program I would be a lot more hesitant to tell you that (#3). |
| Subtheme: Differing opinions   - I would never go into somewhere expecting somebody to think exactly like I do so I mean I’m open to listening to a point of view that I haven’t considered just as I would hope that everyone else would be open to my point of view. There’s no right or wrong it’s just that we’re expressing and I think there could be value in both opinions (#1). - I’ll agree just to disagree with somebody else like I won’t argue and disrupt the whole group, I’ll just, we can talk but I’m not going to argue like you disagree, OK let’s move on (#2). |
| Subtheme: Direct questioning   - I think it’s left up to the group members to know, to pick up on whatever that person is giving you, you know what I’m saying? There are some people that are shy, there are some people who will not speak unless you call on them and there’s other people like “why is he calling on me?” (#2). - I have something to say. I would say seriously I would feel quite comfortable because this is a learning process so I don’t mind sharing (#2). - At first I would probably be kind of embarrassed about it a little bit but I would still share it anyway if I had an opinion (single). |
| Subtheme: Having a process for soliciting opinions   - Turn to that person and ask them, do they have anything they want to share or, if they are uncomfortable, maybe if you could go around the room and ask everybody to share and, probably when it gets to them, they’ll feel more comfortable… Going around, yeah, that’s a little easier than pointing somebody out or putting the spotlight on somebody that is not comfortable. They’ll be ready, especially if you say, we’re going to go around the room, and just say, which side would like to start, and somebody will probably raise their hand (single). - I would suggest always having like a structure as to how do you make sure that everyone’s able to say what they want to say… I think a lot of times it’s hard… jumping in the conversation when you have two physicians that are just like going at it, sometimes it hard to… butt in (#3). |
| Subtheme: Allow people to pass on questions   - If you tell them we’re about to go around the room even if they want to they can say pass and you can go to the next person (single). |
| Subtheme: Allow written submission of thoughts   - Just throw the questions out there and see who wants to answer it, or ask them if they would even like to write it down and submit it, maybe they feel more comfortable that way. Some people might not want to speak out in a group (single). |
| **Theme: Meetings** |
| Subtheme: Supply materials to take notes   - Letting them know that you have paper and pen over here if you want to write anything down, you know if you come up with a thought and somebody else is speaking, you don’t want to lose your train of thought you might want to write it down real quick. So yeah I would say have the pen and paper handy right at the beginning (single) |
| Subtheme: Multiple meetings to build comfort   - A group should meet more than once and the more they met the more spontaneous and comfortable people would be so I would encourage several meetings (#3). - There should be several of these kinds of meetings relative to the level of disease that the patients have because at first people don’t even know what questions to ask and then as they get into more symptomatology and more problems they have frustrating questions that don’t seem to have answers and it grows (#3). - *Acknowledging logistical difficulties with this:* I can see the big picture. These are busy people. You don’t want to spend a lot of time at this right? (#3). |
